# Hairy Root Induction in *Helicteres isora* L. and Production of Diosgenin in Hairy Roots

**DOI:** 10.1007/s13659-014-0011-9

**Published:** 2014-04-09

**Authors:** Vinay Kumar, Dnyanada Desai, Varsha Shriram

**Affiliations:** 1 Department of Biotechnology, Modern College, Ganeshkhind, Pune, 411 016 India; 2 Department of Botany, Prof. Ramkrishna More College, Akurdi, Pune, 411 044 India

**Keywords:** Diosgenin, *Helicteres isora*, Hairy roots

## Abstract

Mature seeds of *Helicteres isora* L. were collected from seven geographical locations of Maharashtra and Goa (India) and evaluated for diosgenin (a bioactive steroidal sapogenin of prime importance) extraction and quantification. Chemotypic variations were evidenced with diosgenin quantity ranging from 33 μg g^−1^ seeds (Osmanabad forests) to 138 μg g^−1^ (Khopoli region). Nodal and leaf explants from in vitro-raised seedlings were used for callus and *Agrobacterium*-mediated transformation, respectively. Compact, hard, whitish-green callus (2.65 g explant^−1^) was obtained on MS + 13.32 μM BAP + 2.32 μM Kin after 30 days of inoculation. Various parameters including types of explant and *Agrobacterium* strain, culture density, duration of infection and various medium compositions were optimized for hairy root production. *A. rhizogenes* strain ATCC-15834 successfully induced hairy roots from leaf explants (1 cm^2^) with 42 % efficiency. Transgenic status of the roots was confirmed by PCR using *rol*B and *Vir*D specific primers. Hairy roots showed an ability to synthesize diosgenin. Diosgenin yield was increased ~8 times in hairy roots and ~5 times in callus than the seeds of wild plants. Enhanced diosgenin content was associated with proline accumulation in hairy roots. This is the first report on induction of hairy roots in *H. isora*.

## Introduction

Diosgenin is a naturally occurring highly bioactive steroidal sapogenin belonging to the group of triterpenes and heavily used in pharmaceutical industry for commercial steroid production [1–3]. Diosgenin is used as an intermediate in the synthesis of corticosteroids, sex hormones, oral contraceptives as well as other steroids via hemisynthesis [4, 5]. In spite of the introduction of new precursors like solasodine, hecogenin and tigogenin for steroidal drug synthesis, diosgenin remains the major precursor. Diosgenin is used in traditional medicine as an anti-hypercholesterolemia, anti-hypertriacylglycerolemia, anti-diabetes and anti-hyperglycemia agent [6–8]. Diosgenin has been reported to show anti-proliferative and pro-apoptotic actions as a chemopreventive and therapeutic agent against various cancers [2, 9] and to prevent cardiovascular diseases [10]. Diosgenin also play an important role in control of cholesterol metabolism [11] and has shown estrogenic effects on mammary epithelium of ovariectomized mouse [12].

*Dioscorea* species are traditionally dominant source of diosgenin and related steroidal saponins [13]. However, overexploitation has led to the rapid depletion of these species and *Dioscorea deltoidea*, the richest source of diosgenin has subsequently become an endangered species, besides sharp shortage of diosgenin in pharmaceutical industry [13, 14]. In India, commercial production of steroidal drugs is totally based on diosgenin [13]; however, the annual production (30 ton) is well short of required (150 ton) and therefore relies on imports, which emphasizes the need to enhance diosgenin production. Therefore, it is crucial to search new and alternative sources of diosgenin and to develop strategies for enhanced production without harming the plant species [15].

*Helicteres isora* L. (Sterculiaceae), commonly known as East Indian Screw Tree has been reported as a source of diosgenin and a major advantage this plant holds is that unlike many other sources, diosgenin is not admixed with other steroidal sapogenins in this plant and therefore makes it easy to isolate it in pure form [16]. One more advantage lies in the abundant availability of *H. isora* throughout India. However, the diosgenin content in this plant has been reported low and needs considerable enhancement prior to its use as a commercial source of diosgenin [16]. All these facts make *H. isora* a preferred candidate for exploration as a source of diosgenin and use of plant cell cultures and genetic transformation approaches for its stimulated production.

We are reporting herein, for the first time, screening of *H. isora* seeds of different geographic locations in Western Ghats regions of Maharashtra and Goa (India) for diosgenin production, identification of elite chemotype, induction of hairy roots and their evaluation for diosgenin production.

## Results and Discussion

### Selection of *H. isora* Chemotype with Highest Diosgenin

**Fig. 1:**
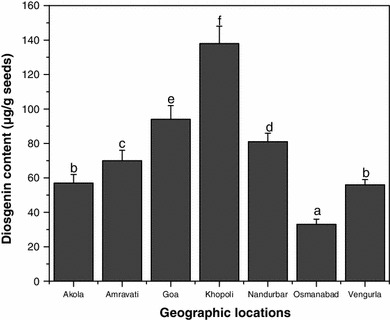
Diosgenin content in seeds collected from various geographical locations in Maharashtra (India). Each value represents the mean of three replications ± standard error. The *bars* with different letters are significantly different from each other at *P* ≤ 0.05 according to Duncan’s Multiple Range Test

Notable eco-geographical variations were evidenced in terms of diosgenin content extracted from seeds of *H. isora* growing in seven regions of Maharashtra and Goa (Fig. 1). Amongst all the samples, the seeds from Khopoli region plants exhibited highest diosgenin (138 μg g^−1^ seeds) content followed by Goa and Nandurbar, whereas the samples collected from the forests in Osmanabad district showed lowest diosgenin (33 μg g^−1^ seeds). The seeds from Khopoli region were therefore selected and used for further experiments. This is the first attempt to assess whether genetic and environmental factors influenced the diosgenin content in *H. isora* seeds collected from different geographical locations of Maharashtra and Goa. Previously, Taylor et al. [17] reported significant eco-geographical variations with respect to diosgenin content in Fenugreek accessions from Canada.

### Callus Production

**Fig. 2:**
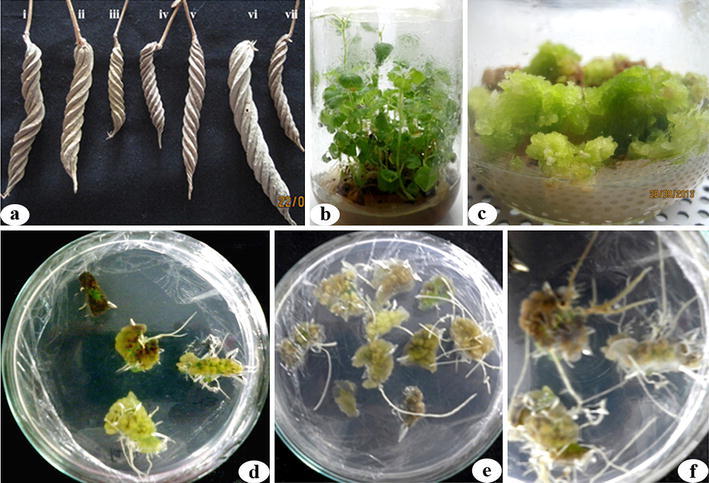
**a** Pods collected from various locations (i: Akola; ii: Amravati; iii: Goa; iv: Khopoli; v: Nandurbar; vi: Osmanabad and vii: Vengurla). **b** In vitro germinated seedlings. **c** 1 Month old callus of *H. isora* obtained on MS + 13.32 μM BAP + 2.32 μM Kin. **d**–**f** Induction and proliferation of hairy roots from leaf explants

Seeds of Khopoli region were germinated in vitro (Fig. 2a, b) following the method described earlier by our group [18] and compact, hard, whitish-green callus (2.65 g fresh weight per nodal explant) was obtained on MS medium containing 13.32 μM BAP + 2.32 μM Kin after 30 days of inoculation (Fig. 2c).

### Hairy Root Induction and Proliferation

**Fig. 3:**
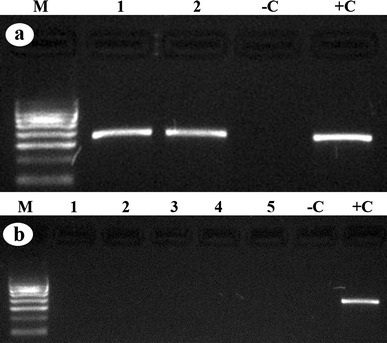
**a** PCR amplification of 423 bp fragment of *rol*B gene using hairy root derived genomic DNA. *Lane M* 100–500 bp molecular weight marker; *Lane 1* and *2* hairy root lines; *Lane* −*C* DNA from non-transformed roots (negative control); *Lane +CAgrobacterium rhizogenes* DNA (positive control). **b** Electrophoregram showing absence of *Vir*D gene (438 bp) in hairy root lines 1–5, *lane −C* DNA from non-transformed roots (negative control), +C: *Agrobacterium rhizogenes* DNA (positive control), *Lane M* 100–500 bp molecular weight marker

For hairy root induction process, selection of *A. rhizogenes* strains, type of explants and growth medium compositions are crucial components. In present investigation, various parameters were optimized for efficient *Agrobacterium*-mediated transformation. Two *A. rhizogenes* strains, ATCC-15834 and MTCC-534 were used for genetic transformation of *H. isora*; however, MTCC-534 failed to induce hairy roots either from leaf or nodal explants. On the other hand agropine type *A. rhizogenes* strain ATCC-15834 successfully transferred its T-DNA to plant tissues and induced hairy roots. ATCC-15834 has been reported as a most widely used *A. rhizogenes* strain owing to its strong induction ability [19–22] and the variation in hairy root induction could possibly be attributed to disparity in virulence of different *A. rhizogenes* strains [23], our results are in accordance with these findings. Amongst two explants, leaves gave superior response than nodes. The choice of plant material is crucial for successful transformation with *A. rhizogenes* [24] and usually, transformation of young tissues gives the best results [19]. Our observations are in conformity of these findings and third and fourth young leaves from apices were found most responsive for transformation. The optimal time for bacterial infection of leaves was observed to be 40 min. The optimal bacterial density for transformation was found to be at 0.8 OD. Two days co-cultivation period was found best for *H. isora* transformation (detailed data not shown). Hairy roots started appearing on the cutting-edges of leaves and at the point of injection on the leaf fragments (Fig. 2d), but not on nodal explants, after 3 weeks of inoculation on MS medium containing 250 mg L^−1^ cefotaxime and 30 g L^−1^ sucrose in dark conditions with 42 % frequency. The growth medium is considered as crucial component of hairy root induction and growth [21]. Various modifications in MS basal medium- i) reducing sucrose content from 30 to 20 g L^−1^; (ii) excluding MS vitamins and including B5 vitamins; (iii) reducing basal strength to half (½-MS); (iv) reducing basal strength to one-fourth (¼-MS) were tried for optimal hairy root production. Amongst those, ¼-MS was found best for hairy root growth, which further proliferated upon their subculturing onto the same medium and incubated at 16 h photoperiod with 25 μmol m^−2^ s^−1^ light intensity (Fig. 2e, f). Hairy root cultures were identified on the basis of their morphology and their genetic nature was further confirmed using PCR amplification of *rol*B gene in the hairy root lines using gene specific forward and reverse primers. Two hairy root lines were selected for PCR confirmation, whereas *A. rhizogenes* served as positive control and DNA from non-transformed seedling roots served as negative control. Both the lines showed presence of 423 bp *rol*B amplified products (Fig. 3a, lane 1 and 2), indicating the integration of T-DNA of *A. rhizogenes* in *H. isora.* This gene has been widely used and advocated for confirmation of *A. rhizogenes*-mediated transformation in hairy roots of various plant species [19–21, 24]. PCR analysis was also carried using *Vir*D gene-specific primers to check the contamination of hairy roots with residual *Agrobacterium*. Results (Fig. 3b lanes 1–5) clearly revealed the absence of contamination of hairy roots with *Agrobacterium* strain. *Vir*D gene was not detected in non-transformed root (Fig. 3b lane −c).

### Diosgenin Production by Callus and Hairy Root Cultures

Diosgenin was detected from crude extracts of seeds of wild plants growing in Khopoli region and compared with the leaves and non-transformed roots of in vitro germinated seedlings, 1 month old callus and 6 weeks old hairy root cultures of *H. isora* using HPTLC. The diosgenin content was significantly higher in hairy roots followed by callus. Callus cultures showed 633 μg diosgenin g^−1^ callus mass as compared with 127 μg diosgenin g^−1^ seeds, indicating five time more diosgenin in callus than seeds of wild plants. However, leaves of in vitro raised seedlings showed significantly lower diosgenin concentration than seeds. Four hairy root lines were independently evaluated for diosgenin content and these lines showed variation in diosgenin content, with lowest of 995 μg g^−1^ hairy roots and highest of 1034 μg diosgenin g^−1^ hairy roots (Table 1). Similar to our findings, enhanced secondary metabolites production has been reported in callus [25] and hairy root cultures [20, 22, 25, 26]. One of interesting findings was that hairy roots accumulated significantly higher amount of proline than non-transformed in vitro roots (Table 1). Proline is a multifunctional proteinogenic amino acid which is known to accumulate in plants in response to biotic and abiotic stresses [27]. This stimulated proline may be attributed to the bacterial infection of explants excised from in vitro raised seedlings. Higher amount of proline over non-transformed roots has been reported previously in hairy roots of *Capsicum frutescens* [28].

**Table 1:** Diosgenin and proline content in seeds, callus, non-transformed leaves and roots of in vitro germinated seedlings and hairy root lines of *H. isora*

Sample	Diosgenin content (µg g^−1^ FW)	Proline content (µmol g^−1^ FW)
Seeds (control)	127 (100)	4.14 (100)
Callus	633 (498)	4.68 (113)
Non-transformed leaves	70 (55)	4.30 (104)
Non-transformed roots	129 (102)	4.57 (110)
Hairy root lines		
T_1_	1008 (794)	28.20 (681)
T_2_	989 (779)	27.5 (664)
T_3_	1034 (814)	28.3 (684)
T_4_	965 (760)	27.4 (662)

T_1–4_: hairy root lines, values in parenthesis shows the percentage increase in diosgenin and proline contents by considering diosgenin and proline content in control non-transgenic seeds as 100 %, respectively

Though *H. isora* has been reported as a source of diosgenin, its content needs to be increased before large scale production from this plant. This is the first report on enhanced diosgenin production in *H. isora* cell and hairy root cultures. Owing to its tremendous usage in pharmaceutical industry and market values, diosgenin is one of the most sought after secondary metabolites of plant origin and therefore various methods are being worked out and reported for its enhanced production in plant tissues including plant cell and suspension cultures [25, 29], via implying abiotic and biotic stresses or elicitors [30] and microbial infections [1]. Narula et al. [30] reported stimulated diosgenin production under CuSO_4_-induced metal stress in *Dioscorea bulbifera* cultures. Similarly, Li et al. [1] successfully enhanced the diosgenin production in *Dioscorea zingiberensis* cell cultures using endophytic fungal infection. The effect of ethylene was also studied on callus cultures of *Trigonella foenum*-*graecum* and authors reported 195 % increase in callus diosgenin content at 25 ppm ethephon (an ethylene releasing compound) treatment [31]. Binesh and Gnanam [25] also reported increased diosgenin in hairy root cultures of *T. foenum*-*graecum.* Hairy roots have various advantages including high growth rate, more genetic stability than callus and suspension cultures, grows well on hormone free media and have been proved very efficient for producing noticeably higher secondary metabolites.

Present study holds significance, as this plant may serve as a good alternative source of diosgenin since it is not admixed with other sapogenins and therefore is easier to isolate from *H. isora*, besides traditional sources of diosgenin, the *Dioscorea* species are under constant pressure and therefore depleting rapidly. The hairy roots of *H. isora* have shown promising results in terms of significant yields of diosgenin and have potential for being scaled-up further for diosgenin production.

## Experimental Section

### Plant Material

Mature pods of *H. isora*, growing in forest areas of seven different geographic regions in Maharashtra including Akola, Amaravati, Khopoli, Nandurbar, Osmanabad, Vengurla and Goa (Fig. 2a) were collected and samples were authenticated at Anantrao Pawar College, Pune and specimen voucher (No. APCP/21/2012-13) was submitted. Seeds from the mature pods were separated and used for further study.

### Extraction and Quantification of Diosgenin

Mature seeds (2 g) separated from pods were finely powdered and extracted with 120 mL of methanol for 5–6 h followed by concentration in vacuo at 40 °C. 25 mL of 15 % methanolic-HCl was added and reflux for 2 h. Acidic solvent was removed under vacuum to dryness. Residue was extracted three times with chloroform and moisture was removed from extract using anhydrous sodium sulfate. Finally solvent was removed completely to get final extract. The callus and hairy root extracts were also prepared in same manner.

For preliminary screening, diosgenin content was estimated spectrophotometrically from the seeds collected from all the seven geographical locations to select elite chemotype with higher diosgenin content as described, based on color reaction of diosgenin with perchloric acid and by referring to a standard curve of diosgenin (Sigma-Aldrich, Germany) [32]. Diosgenin quantification of crude extracts wild type seeds, callus and hairy roots obtained from Khopoli seeds was carried out on a high performance thin layer chromatography (HPTLC) system (CAMAG, Switzerland) comprising of a Linomat-5 applicator and CAMAG TLC densitometer scanner analysis. Stationary phase was silica gel 60 F_256,_ 20 × 10 cm TLC plate (Merck), toluene:chloroform:ethyl acetate (8:8:2) was used as mobile phase and anisaldehyde as a developing reagent. Chromatographs were evaluated by software (winCATS planar chromatography manager) to detect the presence of and quantify diosgenin against the standard.

### Callus Production

Seeds of Khopoli region plants were germinated in vitro as described earlier [18]. Briefly, seeds were treated with concentrated H_2_SO_4_ for 4–5 min to break seed coat followed by washing with 1.0 % Tween-20 for 5 min and three rinses with distilled water. Surface sterilized seeds were germinated on MS medium [33] containing 30 g L^−1^ sucrose, 8 g L^−1^ agar (HiMedia, Mumbai, India) and pH 5.8. Cultures were maintained at 25 ± 2 °C in dark for 4 days and then transferred to 16 h day^−1^ photoperiod at 25 μmol m^−2^ s^−1^ light intensity. Nodal explants (1–1.2 cm) excised from 3 to 4 weeks old in vitro raised seedlings were used for callus formation on MS medium supplemented with 13.32 μM BAP + 2.32 μM Kin.

### Establishment of Hairy Root Cultures

*Agrobacterium rhizogenes* strains ATCC-15834 (NCIM, NCL, Pune) and MTCC-534 (IMTECH, Chandigarh) were grown for 48 h in yeast extract mannitol (YEM) medium. Leaf (1 cm^2^) explants and nodal segments from in vitro raised plants were either immersed or punctured with hypodermic needles attached to a syringe containing overnight grown bacterial suspensions of both *Agrobacterium* strains separately (OD_600_ = 0.5–0.8) for 10–50 min with continuous shaking and were then dried with sterilized Whatman No. 1 filter papers to remove excess bacteria and co-cultivated on solidified hormone free MS media for 1–5 days in dark conditions. One hundred explants were used per experiment and each experiment was carried out in triplicate. The explants were given a wash with 250 mg L^−1^ cefotaxime to kill the residual *Agrobacterium* and the cultures were then transferred to MS medium containing 250 mg L^−1^ cefotaxime, 30 g L^−1^ sucrose and 8 g L^−1^ agar for further growth in complete darkness 25 ± 2 °C for 3 weeks. After this, the hairy root lines were maintained by subculture of 3–5 cm long pieces of roots on the same medium on 16 h day^−1^ photoperiod at 25 μmol m^−2^ s^−1^ light intensity.

### PCR Confirmation of Hairy Roots

Genomic DNA was extracted using CTAB method [34] from young hairy root lines separately, as well as control (non-transformed roots of in vitro germinated seedling), while the Ri-plasmid of *A. rhizogenes* was used as a positive control [35]. Integration of the *rol*B gene into the plant genome was confirmed by PCR analysis [24]. The specific primers for extending 423 bp fragment of nucleotides *rol*B gene were designed and their homology checked through the BLAST search. The primers consisted of nucleotides with the sequence of *rol*B1: 5′-GCTCTTGCAGTGCTAGATTT-3′ and *rol*B2: 5′-GAAGGTGCAAGCTACCTCTC-3′ for the forward and reverse, respectively [24]. PCR analysis was carried out using a GeneAmp PCR System (Applied Biosystems, USA) to confirm the presence of a 423 bp *rol*B gene fragment in the putative transformants. Amplification conditions for *rol*B gene were 1 cycle at 95 °C for 5 min followed by 35 cycles of amplification (95 °C for 30 s, 56 °C for 30 s, 72 °C for 1 min) followed by a final extension step at 72 °C for 10 min. To confirm the transgenics free from any contamination with residual *Agrobacterium*, *Vir*D gene specific primers, forward 5′-ATGTCGCAAGGCAGTAAGCCC-3′ and reverse 5′-GGAGTCTTTCAGCATGGAGCAA-3′ were used and amplification conditions were 1 cycle at 94 °C for 2 min followed by 35 cycles of amplification (45 s at 94 °C, 40 s at 55 °C and 45 s at 72 °C) and 1 cycle at 72 °C for 10 min to give a 438 bp product [36]. The electrophoresis of the PCR products was performed on 2 % agarose gel spiked with ethidium bromide. The gel was visualized under UV light and photographed using GelDoc XR documentation system (BIO-Rad, USA).

### Estimation of Proline from Callus and Hairy Roots

Samples (500 mg) were homogenized in 5 mL 3 % sulfosalicylic acid using a mortar and pestle and centrifuged at 12,000×*g* for 20 min. Two mL aliquot of supernatant was mixed with equal volumes of glacial acetic acid and acid ninhydrin reagent followed by boiling of reaction mixture in water bath at 100 °C for 30 min. After cooling the reaction mixture, 6 mL toluene was added and after thorough mixing, the chromophore containing toluene was separated and absorbance was recorded at 520 nm against toluene blank [37].
